# *Rev-erb*α Knockout Reduces Ethanol Consumption and Preference in Male and Female Mice

**DOI:** 10.3390/ijms23095197

**Published:** 2022-05-06

**Authors:** Yasmine Al-Sabagh, Hayley Hope Allyssa Thorpe, Bryan William Jenkins, Shahnaza Hamidullah, Malik Asfandyaar Talhat, Cara Beth Suggett, Cristine Joelle Reitz, Mina Rasouli, Tami Avril Martino, Jibran Younis Khokhar

**Affiliations:** Department of Biomedical Sciences, Ontario Veterinary College, University of Guelph, Guelph, ON N1G 2W1, Canada; yalsabag@uoguelph.ca (Y.A.-S.); thorpeh@uoguelph.ca (H.H.A.T.); bjenki01@uoguelph.ca (B.W.J.); shamidul@uoguelph.ca (S.H.); mtalhat@uoguelph.ca (M.A.T.); csuggett@uoguelph.ca (C.B.S.); cristine.reitz@utoronto.ca (C.J.R.); mrasouli@uoguelph.ca (M.R.)

**Keywords:** alcohol, circadian rhythms, *REV-ERB*α, *NR1D1*, addiction

## Abstract

Alcohol use is a contributor in the premature deaths of approximately 3 million people annually. Among the risk factors for alcohol misuse is circadian rhythm disruption; however, this connection remains poorly understood. Inhibition of the circadian nuclear receptor REV-ERBα is known to disrupt molecular feedback loops integral to daily oscillations, and impact diurnal fluctuations in the expression of proteins required for reward-related neurotransmission. However, the role of REV-ERBα in alcohol and substance use-related phenotypes is unknown. Herein, we used a *Rev-erb*α knockout mouse line and ethanol two-bottle choice preference testing to show that disruption of *Rev-erb*α reduces ethanol preference in male and female mice. *Rev-erb*α null mice showed the lowest ethanol preference in a two-bottle choice test across all genotypes, whereas there were no ethanol preference differences between heterozygotes and wildtypes. In a separate experiment, alcohol-consuming wildtype C57Bl/6N mice were administered the REV-ERBα/β inhibitor SR8278 (25 mg/kg or 50 mg/kg) for 7 days and alcohol preference was evaluated daily. No differences in alcohol preference were observed between the treatment and vehicle groups. Our data provides evidence that genetic variation in *REV-ERB*α may contribute to differences in alcohol drinking.

## 1. Introduction

Alcohol use is a contributing factor in the premature deaths of approximately 3 million people annually [1]. It is estimated that 23.9% of US citizens engage in binge drinking (4–5 or more drinks during one occasion) and 5.8% in heavy drinking (binge drinking for 5 or more days in a 30-day period), thus contributing to profound morbidity and mortality levels [2]. Those with perturbed circadian cycles, such as shift workers [3], adolescents [4], and frequent trans-meridian travelers [5,6], have greater odds of hazardous alcohol use than the general population. Similarly, chronic alcohol users and those in remission experience circadian rhythm and sleep disturbances, which may also contribute to the risk of relapse [7]. Collectively, these observations suggest that circadian desynchrony shares a reciprocal relationship with adverse alcohol consumption patterns and is a risk factor in problematic alcohol consumption. 

Clinical and preclinical studies illustrate that alcohol use behaviors are linked with variations in genes of the cellular circadian mechanism. Briefly, the positive arm of the circadian mechanism involves Complex Circadian Locomotor Output Cycles Kaput (CLOCK) or its paralogue Neuronal Per-Arnt-Sim Domain Protein 2 (NPAS2), which heterodimerize with Brain and Muscle Arnt-like proteins (BMAL1, BMAL2) to drive transcriptional regulation of gene expression through binding enhancer elements. This in turn drives the expression of proteins that act as feedback regulators of CLOCK-NPAS2/BMAL, including the primary negative feedback loop proteins Cryptochrome (CRY) and Period (PER1, 2, and 3), and the accessory proteins REV-ERBα (otherwise known as Nuclear Receptor Subfamily 1 Group D Member 1). The 24 h day and night cycling of the circadian mechanism and its effects on organ pathophysiology have been well reviewed (e.g., [8,9,10,11,12]). Important to this study, polymorphisms of *CLOCK/NPAS2* [13,14], *BMAL* [15,16], *PER* genes [15,17,18,19,20,21,22], and *REV-ERBα* [23] predict alcohol use phenotypes in humans independently or co-morbidly with other conditions (e.g., depression, stress). Mechanistically, elimination or downregulation of *Clock* [24], *Per1* [21,25], and *Per2* [22,25,26,27] is associated with changes in ethanol consumption patterns and/or sensitivity in rodent models of drinking behavior. Meanwhile, global *Clock* [24], *Per1* [21,25], and *Per2* [22,25] deficiency increases alcohol self-administration and preference; a triple knockdown of *Per1*, *Per2*, and *Clock* localized to the nucleus accumbens reduces alcohol consumption in a mouse model of binge drinking [28]. Together, these findings suggest that circadian biology and circadian mechanism gene mutations play an important role in predicting alcohol consumption phenotypes.

Although many preclinical studies have sought to characterize the role of the core circadian genes within the primary feedback loop on ethanol drinking and response [21,22,24,25,26,27], there is a lack of understanding of how key accessory circadian proteins influence alcohol-related behaviors. Factors such as REV-ERBα are of particular interest because they could be important contemporary targets of drug therapies. Given REV-ERBα gene-based correlation with alcohol use [23] and that agonist administration impairs cocaine reward sensitivity [29], it would seem to be an important target in the treatment of alcohol use disorders as well. Moreover, several studies suggest that REV-ERBα directly regulates dopaminergic neurotransmission, which is key to reward response [30]. In wildtype mice, striatal levels of dopamine are lower at subjective dusk than dawn [31] and the nucleus accumbens exhibits daily fluctuations in its expression of tyrosine hydroxylase (TH) and dopamine transporter, which are required for dopamine synthesis and clearance from the synaptic cleft, respectively [32]. However, genetic or pharmacological inhibition of REV-ERBα in mice results in striatal hyperdopaminergia and ablation of *Th* mRNA transcript periodicity in the ventral midbrain [31]. *Rev-erb*α knockout mice also demonstrate circadian-independent behavioral phenotypes indicative of hippocampal dysfunction and elevated dopamine synthesis, turnover, and *dopamine receptor D1* mRNA transcript levels in the hippocampus [33]. Upregulation of genes involved with dopaminergic signaling in *Rev-erb*α null mice is consistent with phenotypes in *Clock* mutant mice that also show hyperdopaminergic behavioral phenotypes and elevated TH levels, as well as enhanced drug reward [34]. Considering the critical role of dopaminergic activity in drug response and addiction, it seems likely that the circadian mechanism factor REV-ERBα is a critical modulator of alcohol-related phenotypes; thus, being a druggable target, it is a compelling focus of investigation.

Given the role of REV-ERBα in the circadian feedback loop [35], that agonist administration impairs drug reward sensitivity [29], and the similarities between REV-ERBα-depleted rodents and *Clock* mutants in the altered expression of genes related dopaminergic signaling [33,34], we hypothesized that functional disruption of *Rev-erb*α would elevate ethanol consumption and preference. In the first phase of this study, we investigated ethanol two-bottle choice preference in *Rev-erbα* null male and female mice. This experiment was complemented with pharmacological manipulation of REV-ERB via daily injections of the REV-ERBα/β antagonist SR8278 [36]. 

## 2. Results

### 2.1. Genetic Deletion of Rev-erbα Decreases Ethanol Preference and Consumption

**Figure 1:**
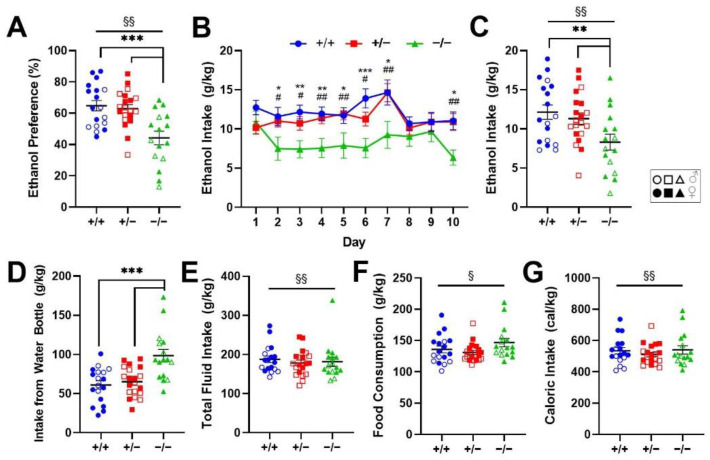
Genetic deletion of *Rev-erb*α reduces ethanol preference and consumption in male and female mice. Preference for 10% ethanol is (**A**) reduced in *Rev-erb*^−/−^ mice relative to *Rev-erb*^+/+^ and *Rev-erb*^+/−^ mice (*p* < 0.001 ***) and elevated in females compared to males (*p* < 0.01 ^§§^). Ethanol intake normalized to body mass is also (**B**) reduced in *Rev-erb*^−/−^ mice compared to the other groups on most testing days (*p* < 0.05 *, < 0.01 **, < 0.001 *** vs. *Rev-erb*^+/+^, *p* < 0.05 ^#^, <0.01 ^##^ vs. *Rev-erb*^+/−^) and (**C**) greater in females compared to males according to daily average consumption (*p* < 0.01 ^§§^). (**D**) Water consumption normalized to body mass was greater in *Rev-erb*^−/−^ compared to the *Rev-erb*^+/+^ and *Rev-erb*^+/−^ groups (*p* < 0.001 ***). Average daily (**E**) total fluid intake (*p* < 0.01 ^§§^), (**F**) food intake (*p* < 0.05 ^§^), (**G**) calorie intake (*p* < 0.01 ^§§^) normalized to body mass was greater in females than males.

To evaluate the role of *Rev-erbα* in voluntary ethanol intake, we employed the two-bottle choice paradigm in a line of *Rev-erbα* knockout mice and compared ethanol preference and consumption across male and female wildtype (*Rev-erb*^+/+^; *n* = 7 males, 11 females), heterozygous (*Rev-erb*^+/−^; *n* = 10 males, 10 females), and knockout (*Rev-erb*^−/−^; *n* = 5 males, 11 females) mice. In the 10-day period following development of stable ethanol preference (less than 15% variance in ethanol preference across 3 consecutive days), a significant effect of genotype was observed on ethanol preference (F_2, 48_ = 11.96, *p* = 6.10 × 10^−5^). Post hoc analysis revealed a significant reduction in ethanol preference in *Rev-erb*^−/−^ (44.18 ± 4.34%) compared to *Rev-erb*^+/+^ (64.83 ± 3.26%, *p* = 2.32 × 10^−4^) and *Rev-erb*^+/−^ counterparts (62.89 ± 2.65%, *p* = 2.10 × 10^−4^; Figure 1A). Consistent with prior research [10,37,38], a significant main effect of sex was also observed (F_1, 48_ = 5.55, *p* = 0.02): ethanol preference was greater in females compared to males regardless of genotype (60.56 ± 3.03% vs. 54.26 ± 3.35%; Figure 1A). However, there were no significant sex × genotype (F_2, 48_ = 0.78, *p* = 0.46), sex × day (F_3.80, 182.51_ = 1.14, *p* = 0.17), or sex × genotype × day (F_7.61, 182.51_ = 0.66, *p* = 0.72) interactions in ethanol preference.

Daily ethanol consumption across the 10-day period was also evaluated according to genotype and sex. Again, a significant main effect of genotype was detected in both daily ethanol (F_2, 48_ = 6.537, *p* = 3.08 × 10^−3^) and water intake from the water-only bottle (F_2, 48_ = 9.857, *p* = 2.59 × 10^−4^), with the knockouts varying significantly from the wildtype and heterozygous groups in both parameters. Average daily ethanol consumption was significantly reduced in *Rev-erb*^−/−^ mice (8.31 ± 1.01 g/kg) compared to *Rev-erb*^+/+^ (12.13 ± 0.92 g/kg, *p* = 5.17 × 10^−3^) and *Rev-erb*^+/−^ (11.31 ± 0.73 g/kg, *p* = 0.01) mice. A significant day × genotype interaction was observed (F_9.26, 222.28_ = 2.34, *p* = 0.01), with post hoc testing revealing lower ethanol consumption in the *Rev-erb*^−/−^ group compared to *Rev-erb*^+/+^ and *Rev-erb*^+/−^ on most testing days (Figure 1B,C). As expected, there was a significant effect of sex on ethanol intake (F_1,48_ = 10.94, *p* = 1.79 × 10^−3^): females voluntarily consumed more ethanol than males (11.81 ± 0.71 g/kg vs. 9.07 ± 0.73 g/kg; Figure 1C). In contrast, water consumption from the water-only bottle was highest in the *Rev-erb*^−/−^ group (98.34 ± 8.06 g/kg), followed by the *Rev-erb*^+/−^ group (65.10 ± 4.15 g/kg, *p* = 9.49 × 10^−4^) and the *Rev-erb*^+/+^ group (60.99 ± 5.27 g/kg, *p* = 6.72 × 10^−4^; Figure 1D). Consistent with these observations, there was a significant main effect of sex on total fluid consumption: females consumed more liquid than males (F_2, 48_ = 9.42, *p* = 3.53 × 10^−3^; 163.35 ± 8.01 g/kg vs. 194.81 ± 6.39 g/kg; Figure 1E). We also observed an effect of day on total fluid consumption (F_4.42, 211.91_ = 6.82, *p* = 3.44 × 10^−9^; data not shown) and greater consumption observed on day 1 compared to days 2, 3, and 4 (*p* < 0.05), and on day 7 compared to most other days (*p* < 0.05), possibly explained by cage cleaning on those days. However, we did not observe any significant main effect of genotype or any interactions on total fluid consumed.

Body mass was also recorded daily and analyzed. There was a significant effect of sex on body mass: weight was lower in females (F_1, 48_ = 218.43, *p* = 1.72 × 10^−19^; data not shown). While there was no significant main effect of genotype on body mass (F_2,48_ = 0.41, *p* = 0.67), there were also significant day × genotype (F_6.37,152.99_ = 3.52, *p* = 2.22 × 10^−3^) and sex × genotype (F_2, 48_ = 4.72, *p* = 0.01) interactions, though neither survived multiple comparison post hoc testing (data not shown). Average daily chow consumption and caloric intake across the 10 days significantly differed according to sex, with females consuming more chow (F_1, 48_ = 5.05, *p* = 0.03; 143.12 ± 4.13 g/kg vs. 128.71 ± 3.60 g/kg) and more calories (F_1,48_ = 8.28, *p* = 5.96 × 10^−3^; 554.78 ± 15.87 Cal/kg vs. 488.25 ± 13.15 Cal/kg) per day on average (Figure 1F,G). There were no significant main effects of genotype (chow consumption: F_2,48_ = 2.00, *p* = 0.15; caloric intake: F_2,48_ = 0.23, *p* = 0.80) or sex × genotype (chow consumption: F_2,48_ = 1.24, *p* = 0.30; caloric intake: F_2,48_ = 0.33, *p* = 0.33) interaction on food consumption patterns, suggesting no effect of genotype on appetitive behavior.

### 2.2. Pharmacological Inhibition of REV-ERBα/β Does Not Affect Ethanol Preference or Intake in Male and Female Mice

**Figure 2:**
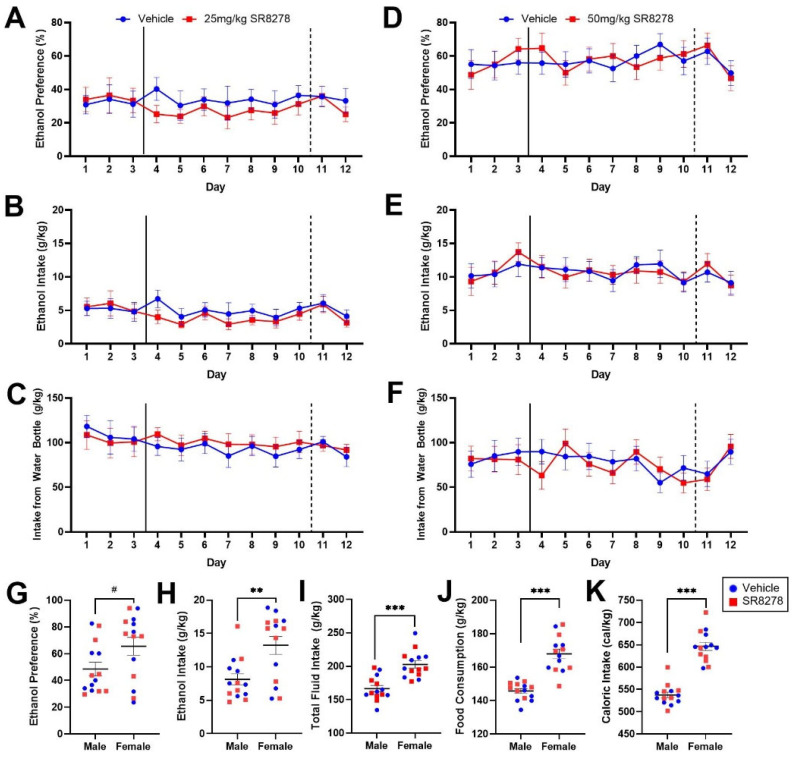
Pharmacological inhibition of REV-ERBα/β with 25 mg/kg (**A**–**C**) or 50 mg/kg (**D**–**K**) of SR8278 did not alter ethanol preference, ethanol intake, or water intake from the water-only bottle in mice. In male mice, 25 mg/kg of SR8278 did not alter (**A**) ethanol preference, (**B**) ethanol intake normalized to body mass, or (**C**) water intake from the water-only bottle normalized to body mass compared to vehicle treatment. In male and female mice, 50 mg/kg of SR8278 did not alter (**D**) ethanol preference, (**E**) ethanol intake normalized to body mass, or (**F**) water intake from the water-only bottle normalized to body mass compared to vehicle treatment. Irrespective of treatment (50 mg/kg SR8278 or vehicle), there was a (**G**) trend toward a significant effect of sex on ethanol preference (*p* = 0.06), with females showing greater average daily (**H**) ethanol intake (*p* < 0.01), (**I**) total fluid intake (*p* < 0.001), (**J**) food intake (*p* < 0.001), and (**K**) caloric intake (*p* < 0.001) compared to males when normalized to body mass. *p* = 0.06 ^#^, *p* < 0.01 **, *p* < 0.001 ***. (**A**–**F**) Solid lines indicate time of onset of SR8278 injection; dashed lines indicate time of cessation of daily injections.

To further evaluate the role of REV-ERBα in voluntary ethanol consumption and ethanol preference, we attempted to recapitulate the findings in the *Rev-erb*α null mouse line using pharmacological means. Using a cohort of male C57Bl/6N mice (*n* = 8/group) with stable ethanol preference in the two-bottle choice paradigm, we administered a vehicle solution or the REV-ERBα/β antagonist SR8278 daily and assessed changes to ethanol preference, liquid consumption, body mass, and food intake. Mice were habituated to vehicle injection for 3 days prior to drug administration, after which vehicle solution or 25 mg/kg of SR8278 was administered for 7 days; recordings continued for 2 post-injection days. On each day, SR8278 or vehicle were administered at zeitgeber time (ZT) 06, when REV-ERBα levels are highest [39]. Overall, there were no significant effects of treatment on ethanol preference (F_4.97, 69.53_ = 1.04, *p* = 0.40), ethanol consumption (F_4.90, 68.62_ = 1.153, *p* = 0.34), water consumption from the water-only bottle (F_4.90, 68.63_ = 0.60, *p* = 0.70), or total fluid intake (F_11, 154_ = 0.317, *p* = 0.98) across the experimental period (Figure 2A–C).

Although not significant, ethanol preference and consumption were marginally lower in male mice treated with 25 mg/kg of SR8278 than the vehicle group across the injection period (Figure 2A,B). We therefore extended these investigations with a naïve cohort of male and female C57Bl/6N mice (*n* = 7/group/sex) injected with 50 mg/kg of SR8278 to assess whether there might be an effect of SR8278 dose or a potential interaction with sex on alcohol drinking. Comparable with our previous results, we observed no significant differences between vehicle and treatment groups in ethanol preference (F_6.17, 148.14_ = 0.99, *p* = 0.46), ethanol intake (F_6.41, 153.87_ = 0.45, *p* = 0.85), or water intake from the water-only bottle (F_5.72, 137.35_ = 1.26, *p* = 0.28) during the injection period (Figure 2D–F). Females consumed more ethanol (F_1, 24_ = 9.66, *p* = 4.79 × 10^−3^; 13.22 ± 1.34 g/kg vs. 8.13 ± 0.83 g/kg), chow (F_1,24_ = 27.72, *p* = 2.12 × 10^−5^; 166.90 ± 2.99 g/kg vs. 146.74 ± 2.34 g/kg), total fluid (F_1,24_ = 23.82, *p* = 5.63 × 10^−5^; 202.78 ± 5.26 g/kg vs. 166.45 ± 5.26 g/kg), and calories (F_1, 24_ = 78.09, *p* = 5.19 × 10^−9^, 644.40 ± 8.26 kcal/g vs. 539.20 ± 8.62 kcal/g) per gram of body weight than males, and there was a trend toward a significant difference in ethanol preference according to sex (F_1, 24_ = 3.881, *p* = 0.06, 65.58 ± 6.62% vs. 48.58 ± 5.02%; Figure 2G–K). In addition, there was a sex × day interaction on total fluid intake (F_11, 264_ = 2.96, *p* = 1.02 × 10^−3^), with females consuming more fluid than males on most days regardless of treatment (*p* < 0.05; data not shown).

## 3. Discussion

Excessive alcohol use is a major public health concern and is especially prevalent among those experiencing circadian disruptions, such as frequent travelers, shift workers, and adolescents [3,4,5,6]. Findings from the present study indicate that expression of *Rev-erbα* (a gene encoding an auxiliary circadian constituent) in mice might promote ethanol preference and self-administration. To our knowledge, this is the first study to causally suggest a function for *Rev-erbα* in patterns of ethanol use. The finding that *Rev-erb*α deletion reduces ethanol self-administration in male and female mice is surprising considering prior observations of REV-ERB modulation on reward response; notably, treatment of mice with the REV-ERBα/β agonist SR9011 was previously shown to attenuate wheel running without affecting overall locomotion [40], and impair cocaine-conditioned place preference [29]. Together with our findings, this may suggest that aberrant REV-ERBα activity in either direction suppresses reward-related behavior, or that the unique actions of dual REV-ERBα/β modulation may differ from the actions of either auxiliary circadian constituent alone.

We hypothesized that *Rev-erb*α knockout mice would consume and prefer ethanol to a greater degree than wildtype mice considering that *Rev-erb*α deficiency results in a hyperdopaminergic profile in reward-related brain regions, an observation consistent with *Clock* mutant mice that also demonstrate greater drug intake, drug self-administration, and reductions in *Rev-erb*α mRNA transcripts [41,42]. However, our results suggest that *Rev-erb*α knockout mice may find ethanol less rewarding or are less motivated to consume ethanol despite their behavioral and neurobiological similarities to *Clock* mutants in other domains. REV-ERBs participate in negative feedback of their expression by suppressing their own transcription [43] as well as transcription of *Bmal1* [35], whose protein product heterodimerizes with CLOCK to transcriptionally upregulate components of the circadian loop, including *Rev-erbs*. Presumably, the core circadian loop would remain intact in *Rev-erb*α^-/-^ mice, unlike *Clock* mutants. While both genetic lines demonstrate some level of disrupted circadian rhythmicity in activity and clock machinery, *Clock* mutants have severe circadian arrhythmia under constant darkness [44] and reduced expression of circadian mRNA transcripts [45,46,47] in the suprachiasmatic nucleus, whereas *Rev-erb*α knockouts show a more modest effect on periodicity [48] and elevated circadian clock machinery transcript levels [35,49,50], including *Bmal1*. Interestingly, whole-brain BMAL1 expression in male mice is negatively correlated with genetic vulnerability to consuming ethanol in a drinking in the dark paradigm [51], and striatal deletion of *Bmal1* enhances daily ethanol consumption in males while decreasing consumption in females [52]. These findings support that reduced ethanol consumption and preference by *Rev-erb*α deletion could be caused by elevated BMAL1 expression. Although more investigations must be conducted to determine the scope of its impact on downstream effector proteins, our findings in combination with others suggest that *REV-ERB*α could be a potential candidate for the development of novel pharmacotherapies targeting ethanol misuse.

Drug response and addiction liability are principally mediated by dopaminergic signaling through mesocorticolimbic pathways. Dopaminergic dysregulation in the rodent brain by REV-ERBα inhibition has been observed through irregular expression of dopamine-related behaviors, elevated dopamine turnover, higher expression of mRNA and protein related to dopamine metabolism, and disrupted dopaminergic cyclicity in the ventral midbrain; these characteristics culminate in hyperdopaminergia in *Rev-erb*α^−/−^ mice [31,33]. Notably, elevating tonic dopamine neurotransmission by optogenetic stimulation of the VTA is known to attenuate self-administration in ethanol-drinking rats [53,54]. However, alcohol response, sensitivity, and reward are influenced directly by multiple neurotransmitter systems, notably at GABA receptors where ethanol acts to produce some of its acute and chronic effects [55]. Two recent studies revealed that *Rev-erbs* affect oscillatory GABAergic patterns in the brain, with *Rev-erb*α disruption elevating tonic GABA currents in the hippocampus and impairing the oscillatory patterns of GABA reuptake proteins in the hippocampus and cortex [56,57]. It is currently unclear if decreased ethanol preference by *Rev-erb*α deletion is primarily due to impaired dopamine activity affecting reward response or by ancillary systems that participate in ethanol response directly, such as GABAergic neurotransmission. Future investigations of this mouse line and vulnerability to other drugs and naturally rewarding substances will help elucidate whether *Rev-erb*α contributes to ethanol vulnerability specifically, or whether expression of this gene is relevant to generalized drug reward.

In our attempt to establish the effect of one such pharmacotherapeutic strategy, treatment of C57Bl/6N mice with 25 mg/kg or 50 mg/kg of the REV-ERBα/β antagonist SR8278 did not recapitulate the decreased ethanol intake and preference observed in our genetic knockout model. Considering the rapid metabolism of SR8278 in the brain following 25 mg/kg i.p. injection [57], it is likely that a once daily injection of SR8278 is insufficient to affect reward-related functions over the course of a day despite administration during peak *Rev-erb*α expression. Moreover, since we did not assess any other behavioral end-points to assess the effects of SR8278, it is possible that the doses tested here were not behaviorally relevant. It is notable that most studies of *Rev-erb*α on behavior and reward exclusively use genetic models, with only one study comparing the genetic model with bidaily intraperitoneal REV-ERBα/β agonist injections [29], and another microinfusing SR8278 directly into the ventral midbrain 4 h prior to behavioral testing [31]. While our expectation that pharmacological inhibition of REV-ERBs would recapitulate the findings of *Rev-erbα* deletion, future investigations may benefit from the use of the agonist previously observed to affect behavior, or utilizing more invasive, direct methods of antagonist delivery. However, it is also possible that *Rev-erb*α expression during development may affect future ethanol drinking patterns, which would not be apparent with pharmacological inhibition restricted to adulthood. In addition, the role of *Rev-erb*β in circadian circuitry has been relatively underexplored compared to *Rev-erb*α, and while *Rev-erb*β is considered functionally redundant to *Rev-erb*α, we cannot discount a unique function of *Rev-erb*β in ethanol intake vulnerability, warranting specific pharmacological manipulation of each target alone in future studies. Lastly, we did not assess drinking microstructure (e.g., bout duration, licks, etc.), which may be assessed in future studies to establish the effects of REV-ERBα/β pharmacological manipulation on alcohol drinking [58].

It is also possible that decreased ethanol preference in our knockout model is due to an intermediate behavioral phenotype. For instance, there could be enhanced sensitivity to the effects of ethanol within the knockout mice, which may mediate intake. As preference for bitter tasting substances has not been characterized in this line, we also cannot discount a potential difference in taste perception according to genotype. However, one previous study found that *Rev-erbα* knockout mice show enhanced consumption of high caloric sweet food but no differences in the intake of the non-caloric sweetener saccharine [59]. This may suggest no differences in sweet palatability according to genotype, although further preference studies that include high caloric sweet alcohol, low caloric sweet alcohol, and unsweetened alcohol could provide insights into genetic vulnerability to consuming high caloric sweet-tasting (e.g., cocktails, coolers) versus bitter-tasting (e.g., hard liquors, beer) alcoholic beverages. Moreover, it would be important to assess whether REV-ERBα disruption impacts the effects of alcohol on righting reflex and other alcohol-related effects. Previous findings using transgenic mice with alterations in other diurnal function-related genes have some effects on alcohol loss of righting reflex (e.g., *Clock*), whereas *Per1* and *Per2* disruption did not impact loss of righting [24,25]. Finally, multiple studies suggest that *Rev-erb*α affects mood, and REV-ERBα disruption diminishes depression- and anxiety-like phenotypes in mice [31,60]. A positive relationship between mood disorders and alcohol use has been well-documented [61], suggesting that *Rev-erb*α knockout mice could show lower ethanol preference than wildtype counterparts due to greater mood resilience. 

In summary, this work establishes a novel causative association between *Rev-erb*α and ethanol preference in a genetic model of alcohol use vulnerability. These findings provide support for previous correlative human studies highlighting *REV-ERB*α polymorphisms in the risk for developing alcohol use disorders. This is the first study to provide evidence that *Rev-erb*α expression may alter drug use vulnerability, possibly by affecting the expression of core clock components, suggesting that *REV-ERB*α could be a future pharmacological target for mitigating problematic substance use.

## 4. Materials and Methods

### 4.1. Ethics Statement

All experiments herein were approved by the University of Guelph Institutional Animal Care and Use Committee (AUP#3922, 3858) and were performed in accordance with the guidelines set forth by the Canadian Council on Animal Care (1993).

### 4.2. Two-Bottle Choice Test

All mice were given ad libitum access to tap water, 10% ethanol, and standard food chow, as in previous studies [42,62,63]. Water and ethanol bottles had stainless steel drinking spouts inserted through grommets on the cage lids. A two-bottle choice paradigm was implemented by rotating the water bottle and ethanol bottle positions daily to prevent a positional preference. Ethanol, water, food consumption, and body weight were measured and recorded daily at ZT06.

### 4.3. Drug Administration

The REV-ERBα/β inhibitor SR8278 was dissolved in a 5:5:90 solution of DMSO:Cremophor:PBS as previously described (Welch et al., 2017). Injection administration began when there was less than 15% variance in ethanol preference across 3 consecutive days (approximately 2–3 weeks of access). Mice were pseudo-randomly assigned to treatment groups such that there were an equal number of low and high ethanol preferers in each group. The mice were habituated to a 10mL/kg intraperitoneal vehicle injection once daily for 3 days prior to drug administration to mitigate the effects of handling and injection stress on ethanol drinking. REV-ERBα/β inhibitor SR8278 was delivered at 25 mg/kg or 50 mg/kg at ZT06. One group continued to receive vehicle injections and the other were given the REV-ERBα/β inhibitor once daily for 7 days. Measurements were continued for 2 days post-injection.

### 4.4. Experiment 1: Genetic Disruption 

Adult (approximately 8 weeks old) male and female *Rev-erb*^+/+^, *Rev-erb*^+/−^, and *Rev-erb*^−/−^ mice (*n* = 5–11/sex/genotype) were obtained from a breeding colony maintained by Dr. Tami Martino at the University of Guelph. Heterozygous founders (B6.Cg-*Nr1d1*^tm1Ven^/LazJ, strain no. #018447) were purchased from Jackson Laboratory (Bar Harbor, ME, USA). Mice were pair-housed in cages divided by perforated metal dividers to ensure mice only accessed their own food, water, and ethanol while also avoiding the confounding effects of social isolation [42,64]. A constant environment was maintained, with an ambient temperature of 21 ± 2 °C, circulating air, and a constant humidity of 50 ± 10%. Mouse cages were housed in custom-built cabinets under controlled conditions to maintain mice on a standard 12 h light (L):12-h dark (D) cycle, with lights on at 10 am (ZT0) and lights off at 10 pm (ZT12). All mice were habituated to the 12:12 LD cycle for 1 week prior to the start of the study period. 

### 4.5. Experiment 2: Pharmacological Disruption 25 mg/kg

C57Bl/6N male mice (*n* = 8/group) were obtained from Charles River Laboratories (Saint Constant, QC, CA) at approximately 8 weeks old. Mice were housed in pairs with perforated metal dividers. They were habituated to the vivarium and handled by experimenters for 1 week before experiment commencement. A constant environment was maintained, with an ambient temperature of 21 ± 2 °C, circulating air, a constant humidity of 50 ± 10% and a 12:12 LD cycle beginning at 7 am (ZT0). SR8278 was administered daily as described above.

### 4.6. Experiment 3: Pharmacological Disruption, 50 mg/kg

C57Bl/6N male and female mice (*n* = 7/group/sex) were obtained from Charles River Laboratories (Saint Constant, QC, CA, USA) at approximately 8 weeks old. In adherence with recently updated housing guidelines by the Canadian Council on Animal Care, mice were singly housed. Otherwise, experimental conditions and drug administration were the same as for Experiment 2. 

### 4.7. Statistics

All data were analyzed using SPSS 26 (IBM, Armonk, NY, USA), and graphs were generated in GraphPad Prism version 9 (GraphPad Software Inc., La Jolla, CA, USA). Ethanol preference, ethanol consumption, water consumption, and body mass data were analyzed across recorded days, whereas daily food consumption and daily caloric intake were averaged across the total experimental period and within experimental phases when appropriate. Ethanol preference was calculated as % Preference = Ethanol intake (g)/Total Liquid Intake (g) × 100%. Ethanol consumption was reported as the amount of absolute ethanol consumed in the 10% ethanol solution. Caloric intake was calculated as the sum of calories from food chow (3.3 kcal/g) and calories from 100% ethanol (7 kcal/g). Daily ethanol intake, water intake, food intake, and caloric intake were normalized to daily body mass in kilograms. All data are reported at the onset of stable ethanol preference (<15% variance across 3 consecutive days) and are presented as group means ± standard error of the mean. Where appropriate, a univariate ANOVA or repeated measures ANOVA was conducted followed by Bonferroni post hoc testing, with significance determined at *p* < 0.05. 

## Data Availability

The data generated and presented in this study are openly available in Open Science Framework at https://osf.io/uynf6/?view_only=1e7721ea2eee4708ab703524d0a79927.
